# Ferroptosis: a key driver and therapeutic target in the pathogenesis of acute respiratory distress syndrome

**DOI:** 10.3389/fimmu.2025.1567980

**Published:** 2025-07-22

**Authors:** Mingjun Yao, Jinfeng Liao, Zheng Liu, Wei Zhao, Siyuan Song, Xiaobo Huang, Yi Wang

**Affiliations:** ^1^ School of Medicine, University of Electronic Science and Technology of China, Chengdu, China; ^2^ Department of Dermatology, Sichuan Provincial People’s Hospital, University of Electronic Science and Technology of China, Chengdu, Sichuan, China; ^3^ Department of Neuroscience, Baylor College of Medicine, Houston, TX, United States; ^4^ Department of Critical Care Medicine, Sichuan Provincial People’s Hospital, University of Electronic Science and Technology of China, Chengdu, Sichuan, China; ^5^ Translational Clinical Immunology Medicine Key Laboratory of Sichuan Province, Center of Organ Transplantation, Sichuan Academy of Medical Science and Sichuan Provincial People’s Hospital, Chengdu, Sichuan, China

**Keywords:** cell death pathways, immune homeostasis, acute respiratory distress syndrome, ferroptosis, immune cytokines

## Abstract

Acute Respiratory Distress Syndrome (ARDS) is a severe inflammatory lung condition often triggered by infections or sepsis, characterized by diffuse alveolar damage, pulmonary edema, and impaired gas exchange. Despite advances in supportive care, ARDS continues to have a high mortality rate. The pathogenesis of ARDS involves an exaggerated immune response leading to tissue damage and inflammation. Regulatory cell death pathways, particularly ferroptosis, an iron-dependent form of cell death driven by lipid peroxidation and oxidative stress, play a critical role in ARDS progression. Ferroptosis is characterized by the accumulation of lipid peroxides and is regulated by enzymes such as glutathione peroxidase 4 (GPX4) and the system Xc- antiporter. Dysregulation of these pathways exacerbates oxidative stress and tissue damage in ARDS. In the context of ARDS, ferroptosis contributes to the destruction of alveolar and endothelial cells, leading to increased vascular permeability, pulmonary edema, and impaired gas exchange. Immune cells like macrophages and neutrophils, while essential for pathogen clearance, can also contribute to lung injury when overactivated, highlighting the need for therapeutic strategies to modulate ferroptosis. Therapeutic targeting of ferroptosis in ARDS includes the use of antioxidants, GPX4 activators, iron chelators, and inhibitors of lipid peroxidation. These approaches aim to reduce oxidative stress, restore antioxidant defenses, and prevent iron-driven cell death. Future research must address challenges in identifying reliable biomarkers, understanding subphenotype-specific mechanisms, and integrating ferroptosis inhibitors into existing therapeutic frameworks. By targeting ferroptosis, it may be possible to mitigate ARDS severity and improve patient outcomes, offering new hope for the management of this devastating condition.

## 1 Introduction

Acute respiratory distress syndrome (ARDS) is a severe, life-threatening inflammatory condition of the lungs commonly triggered by infections such as bacterial pneumonia, viral pathogens (e.g., influenza and SARS-CoV-2), and systemic sepsis (1). Hallmarked by diffuse alveolar damage, pulmonary edema, hypoxemia, and impaired gas exchange, ARDS frequently necessitates mechanical ventilation in intensive care units (ICUs) (1, 2). Despite advancements in supportive care, infection-driven ARDS continues to have a high mortality rate of 30–50% (3).

The pathogenesis of ARDS involves an exaggerated immune response that damages alveolar structures, disrupts the alveolar-capillary barrier, and promotes excessive inflammation, vascular leakage, and oxygenation failure (4, 5). Immune cells like macrophages and neutrophils, while essential for pathogen clearance, contribute to lung injury when overactivated (6, 7). This highlights the critical need to maintain immune homeostasis to mitigate disease progression (8).

Regulated cell death pathways, such as apoptosis, pyroptosis, necroptosis, and ferroptosis, play pivotal roles in controlling inflammation and maintaining tissue integrity. However, their dysregulation can exacerbate inflammation and tissue damage in ARDS (9, 10). Among these, ferroptosis, an iron-dependent form of cell death driven by lipid peroxidation, oxidative stress, and glutathione depletion, has garnered significant attention (11). In ARDS, ferroptosis contributes to the destruction of alveolar and endothelial cells, thereby exacerbating lung injury (12).

Given the central role of oxidative stress and lipid peroxidation in ARDS, targeting ferroptosis represents a promising therapeutic strategy (13). This review explores the mechanisms of ferroptosis, its role in ARDS progression, and its potential as a target for therapeutic interventions.

## 2 Ferroptosis in infection and ARDS development

Cell death pathways play critical roles in immune homeostasis, infection defense, and inflammation resolution. In ARDS, an imbalance between these pathways contributes to exaggerated immune responses, tissue damage, and disease progression (14) Among these pathways, ferroptosis has emerged as a key player in infection-induced lung injury, offering new therapeutic insights for ARDS management (12).

### 2.1 Ferroptosis: a central player in ARDS pathogenesis

Ferroptosis is a regulated, iron-dependent form of cell death distinguished by oxidative damage and lipid peroxidation. It plays a dual role in infection and inflammation. While ferroptosis helps eliminate infected or damaged cells, its dysregulation exacerbates inflammation, tissue injury, and alveolar-capillary barrier dysfunction in ARDS (15). Understanding the mechanisms of ferroptosis offers opportunities for therapeutic intervention, especially in targeting oxidative stress and iron dysregulation.

### 2.2 Key molecular mechanisms of ferroptosis

Ferroptosis is characterized by the iron-dependent accumulation of lipid peroxides, driven by dysregulated oxidative stress and impaired antioxidant defenses. A central player in this process is glutathione peroxidase 4 (GPX4), an enzyme responsible for neutralizing lipid peroxides. When GPX4 is inhibited or depleted, lipid peroxidation spirals out of control, leading to cell death (16). The system Xc^-^, which imports cystine for glutathione (GSH) synthesis, also plays a crucial role. Dysfunction of system Xc^-^ depletes GSH, further compromising the cell’s ability to counteract oxidative damage (17).

**Figure 1:**
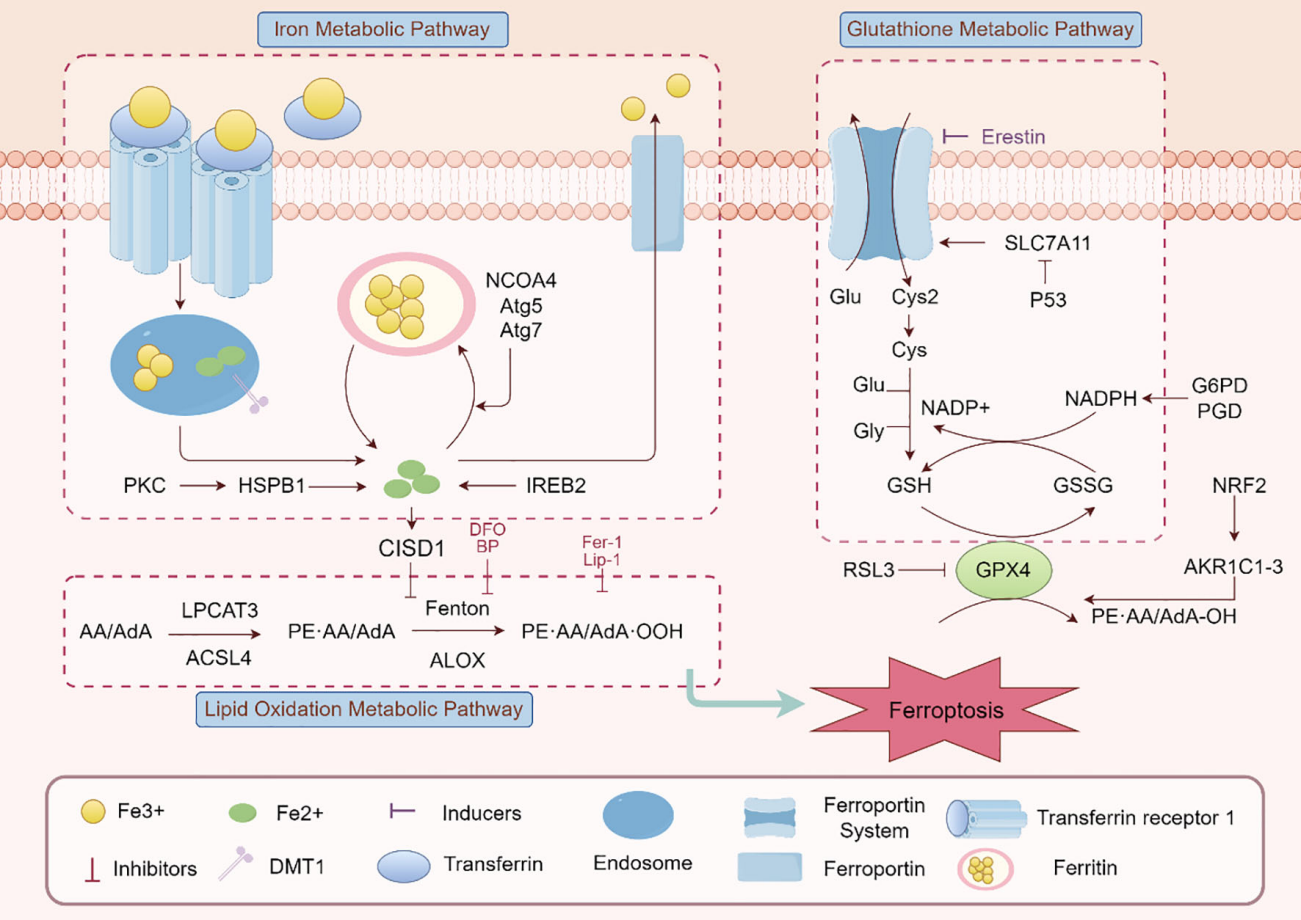
Ferroptosis signaling pathway. The main elements and interactions of ferroptosis, a type of controlled cell death marked by iron-dependent lipid peroxidation, are depicted in this figure. The pathway includes lipid oxidation (e.g., ALOX, LPCAT3), glutathione metabolism (e.g., GSH/GSSG, GPX4), and iron metabolism (e.g., transferrin receptor, ferroportin). Ferroptosis modulation is greatly aided by regulatory proteins like as Nrf2, p53, and SLC7A11. Additionally shown are ferroptosis inducers (like RSL3) and inhibitors (like Fer-1), emphasizing the harmony between pro- and anti-ferroptotic elements.

Iron metabolism is another critical factor in ferroptosis. Excess free iron, often resulting from inflammation-induced disruption of iron homeostasis, generates reactive oxygen species (ROS) through the Fenton reaction (18). These ROS amplify lipid peroxidation, particularly in cell membranes rich in polyunsaturated fatty acids (PUFAs). Enzymes like ACSL4 (acyl-CoA synthetase long-chain family member 4) facilitate the incorporation of PUFAs into membrane phospholipids, increasing their vulnerability to peroxidation (19). Together, these mechanisms underscore the intricate interplay of oxidative stress, lipid metabolism, and iron dysregulation in driving ferroptosis (**Figure 1**).

### 2.3 Triggers and amplifiers of ferroptosis in ARDS

The pathological environment of ARDS provides multiple triggers for ferroptosis, exacerbating lung injury and inflammation. Oxidative stress is a hallmark of ARDS, with elevated ROS levels initiating and propagating lipid peroxidation (20–22). Mitochondrial dysfunction, a frequent feature in ARDS, further contributes to ROS generation and disrupts cellular redox balance, creating a vicious cycle of oxidative damage (22).

Inflammation also amplifies ferroptosis through cytokine-mediated pathways. Pro-inflammatory mediators, such as TNF-α and IL-1β, enhance iron dysregulation by promoting ferritin degradation and releasing free iron into the cytosol. This excess iron accelerates lipid peroxidation, aggravating tissue damage (22). Additionally, systemic inflammation and vascular leakage in ARDS disrupt iron transport and storage mechanisms, further compounding the pro-ferroptotic state (23). These interconnected triggers highlight the multifactorial nature of ferroptosis in ARDS and its significant role in disease progression.

### 2.4 The role of hypoxia in ferroptosis induced ARDS

**Figure 2:**
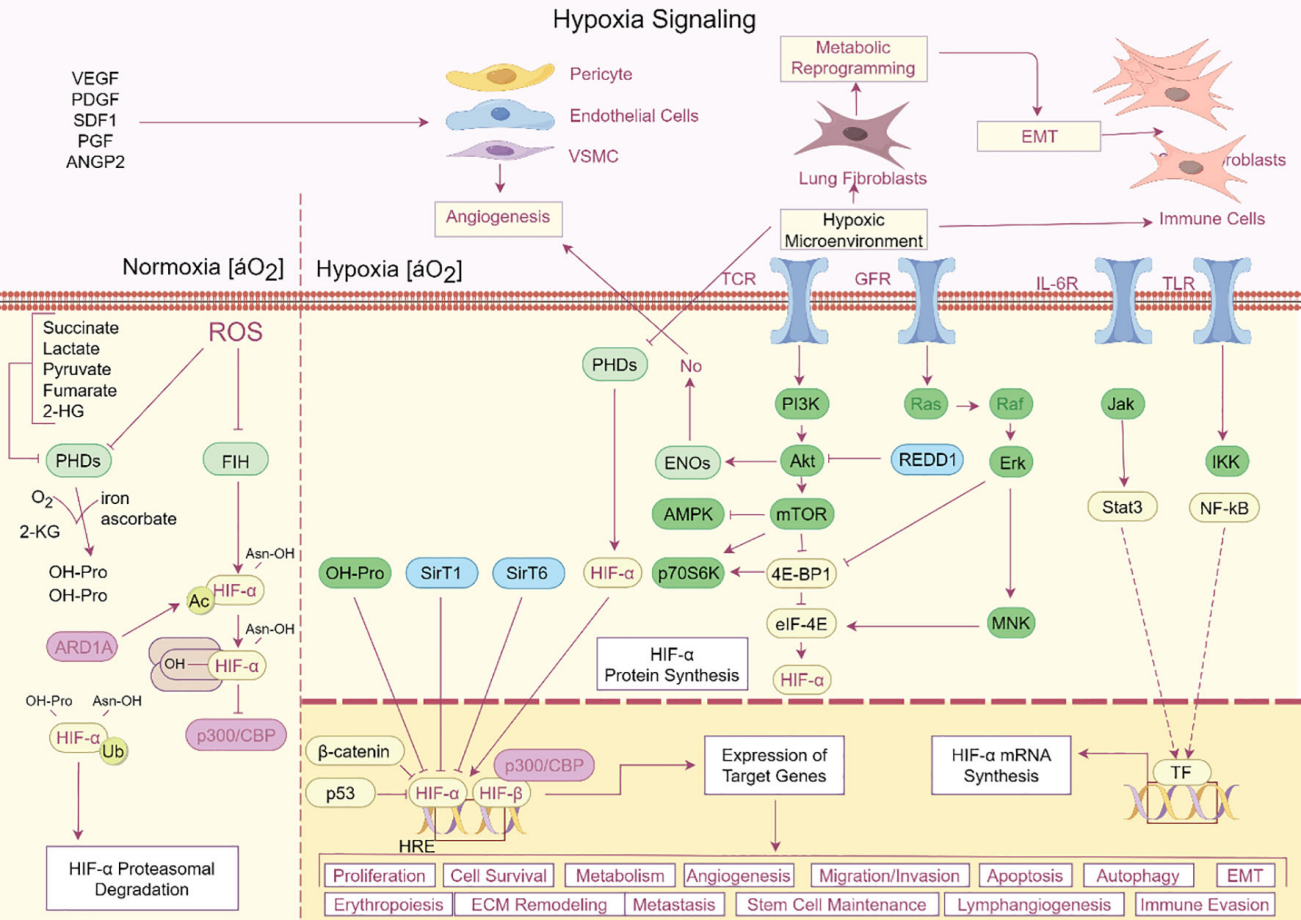
Hypoxia signaling pathway in ARDS. The complex hypoxia signaling cascade and its various impacts on cellular functions are depicted in this image. Oxygen levels fall in hypoxic environments, stabilizing HIF-α, which then dimerizes with HIF-β to bind HRE elements and control the expression of target genes. Numerous biological processes, including as cell division, survival, metabolism, angiogenesis, erythropoiesis, extracellular matrix remodeling, metastasis, and immune evasion, are impacted by this system. HIF-α is further stabilized by metabolic alterations such as increased lactate and succinate synthesis, which block PHDs and FIH. Angiogenesis and immune cell recruitment are encouraged by the upregulation of signaling molecules like VEGF, PDGF, and SDF-1. Hypoxia also affects stem cell maintenance, the epithelial-mesenchymal transition (EMT), and cell migration/invasion. Important regulators that interact with HIF-α to modify cellular responses are also highlighted in the picture, including PI3K, mTOR, AMPK, and NF-kB. This intricate network emphasizes how important hypoxia is in influencing the cellular microenvironment and triggering adaptive reactions in a range of healthy and pathological settings.

**Figure 3:**
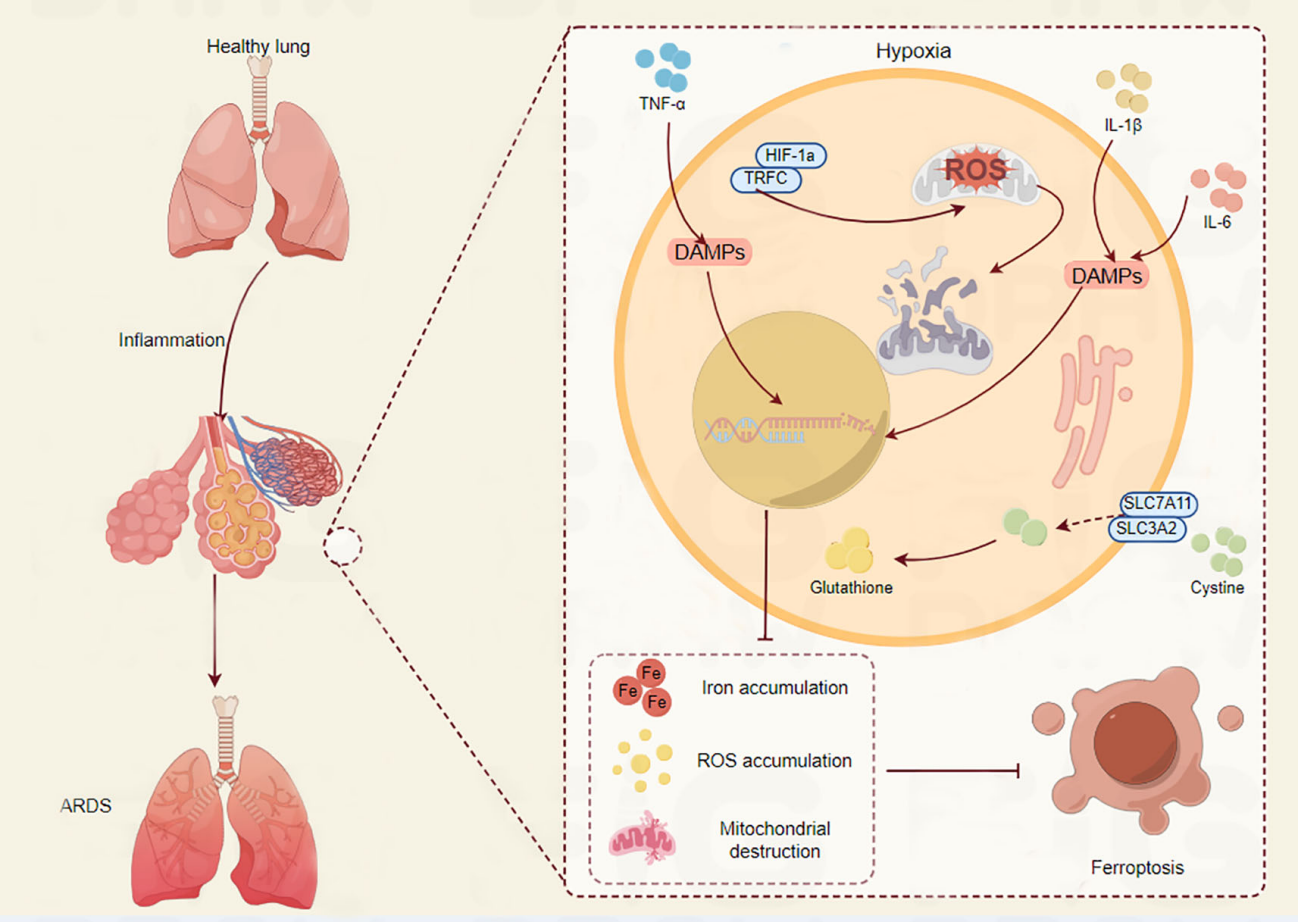
The crucial role of ferroptosis in ARDS progression. The connection between ferroptosis and hypoxia in relation to lung injury is depicted. The lung maintains homeostasis under normal circumstances. On the other hand, TNF-α and IL-1β levels rise during hypoxia, which causes HIF-1α to stabilize. This sets off a series of events that include the release of damage-associated molecular patterns (DAMPs), which fuel inflammation, and the generation of reactive oxygen species (ROS). By raising glutathione levels, the xCT cystine-glutamate antiporter’s components SLC7A11 and SLC3A2 are upregulated in an effort to combat oxidative stress. But this is frequently not enough. The condition is made worse by the buildup of iron and ROS, which ultimately results in the loss of mitochondria. Acute respiratory distress syndrome (ARDS) is a result of these alterations, which ultimately lead to ferroptosis, a type of controlled cell death. The intricate relationship between ferroptosis, inflammation, and hypoxia in lung pathology is depicted in this image.

A common ARDS symptom, hypoxia, worsens lung damage in a number of ways. It is becoming more widely acknowledged that ferroptosis, a newly identified kind of controlled cell death triggered by iron-dependent lipid peroxidation, plays a major role in the pathophysiology of ARDS (24). A defining feature of ARDS is hypoxia, which is brought on by compromised gas exchange as a result of edema and alveolar injury (25). Pulmonary vasoconstriction, endothelial dysfunction, and the production of inflammatory cytokines are among the physiological and pathological alterations brought on by hypoxia. The hypoxia-inducible factor (HIF) pathway, which controls genes related to energy metabolism and iron homeostasis, is activated by the hypoxic environment in ARDS (26). Lung damage may be exacerbated by this pathway’s ability to alter the equilibrium between the oxidative and antioxidant systems (**Figure 2**). In the context of ARDS, recent research has emphasized the link between ferroptosis and hypoxia. Ferroptosis can be facilitated by hypoxia in a number of ways. First of all, ferroptosis may be impacted by the overexpression of genes related to iron metabolism, such as HO-1, caused by the activation of HIF-1α in hypoxic environments (27). Second, oxidative stress brought on by hypoxia can deplete antioxidants such as glutathione, increasing the vulnerability of cells to ferroptosis and lipid peroxidation (28). For instance, via activating the Nrf2/HO-1 pathway and preventing ferroptosis, ferulic acid was demonstrated to mitigate alveolar epithelial barrier failure in sepsis-induced acute lung damage (29). This implies that one possible treatment approach for ARDS patients may be to target ferroptosis to reduce the damage that hypoxia causes to the lungs (**Figure 3**).

### 2.5 Crosstalk of ferroptosis with other cell death

The onset and progression of ARDS are significantly influenced by ferroptosis as well as other types of controlled cell death, including apoptosis, necroptosis, and pyroptosis (30).

Despite being two different types of cell death, ferroptosis and apoptosis have several regulatory mechanisms in common. Iron-dependent lipid peroxidation is the main cause of ferroptosis, which results in the buildup of lipid peroxides and cell death. Apoptosis, on the other hand, is distinguished by the cleavage of certain substrates and the activation of caspases. Recent research, however, has demonstrated that apoptosis and ferroptosis can interact and affect one another. For instance, iron may be released from mitochondria as a result of apoptosis activation, which may subsequently encourage ferroptosis. Furthermore, apoptosis can be attenuated by inhibiting ferroptosis since it lowers the production of pro-apoptotic proteins (31, 32).

The necrosome, a complex made up of receptor-interacting protein kinase (RIPK) 1 and RIPK3, is responsible for regulating necrosis, or necroptosis (33). When necroptosis is triggered, mixed lineage kinase domain-like pseudokinase (MLKL) is phosphorylated, creating holes in the cell membrane and ultimately resulting in cell death (34). Ferroptosis and necroptosis can interact and affect one another, according to recent research. For example, iron may be released from injured cells as a result of necroptosis activation, which may subsequently encourage ferroptosis. The release of damage-associated molecular patterns (DAMPs), which are known to cause necroptosis, can be decreased by inhibiting ferroptosis (35).

Inflammasome activation causes pro-inflammatory cytokines, including IL-1β and IL-18 to be released during pyroptosis, a type of programmed cell death. Ferroptosis and pyroptosis can interact and affect one another, according to recent research. For instance, in deadly polymicrobial sepsis, gasdermin D-mediated pyroptosis can be triggered by lipid peroxidation during ferroptosis. Furthermore, ferroptosis suppression can attenuate pyroptosis by lowering the release of pro-inflammatory cytokines (9).

Numerous important molecular pathways are involved in the crosstalk between ferroptosis, necroptosis, and pyroptosis. The control of lipid peroxidation and iron metabolism is one important mechanism. Cell death may result from lipid peroxidation brought on by iron excess. Enzymes like glutathione peroxidase 4 (GPX4), which lowers lipid hydroperoxides and inhibits lipid peroxidation, control this process. Ferroptosis results from increased lipid peroxidation caused by GPX4 deficiency (36). The tumor suppressor p53 and its downstream targets regulate ferroptosis, which is another significant mechanism. By upregulating genes related to lipid peroxidation and iron absorption, p53 can cause ferroptosis (37). Furthermore, the Nrf2/ARE pathway, which controls redox homeostasis, is essential for preventing ferroptosis in cells. Nrf2 helps sustain cellular viability by transcriptionally activating anti-ferroptotic genes, including SLC7A11 and HO-1 (38).

## 3 Ferroptosis in the development of ARDS

### 3.1 Alveolar epithelial cell damage

The distinct functions of ferroptosis in alveolar type I (AT1) and type II (AT2) epithelial cells have been brought to light by recent research, offering information about possible treatment targets (39).

AT1 cells regulate gas exchange and preserve alveolar shape (40). These cells are especially prone to ferroptosis because of their exposure to oxidative stress and role in maintaining the alveolar-capillary membrane (41). However, the majority of studies focused on AT2 cells. AT2 cells are essential for the synthesis of surfactants and alveolar healing. Particularly in diseases like sepsis and ischemia-reperfusion injury, ferroptosis in AT2 cells has been linked to the pathophysiology of ARDS (42). AT2 cells displayed ferroptotic symptoms, such as mitochondrial contraction and ruptured mitochondrial membranes, in a study on sepsis-induced ALI/ARDS (43). The study also emphasized how regulatory elements like MUC1 suppress ferroptosis by modulating the GSK3β/KEAP1-Nrf2-GPX4 axis. MUC1 has been demonstrated to prevent ferroptosis in AT2 cells by promoting Nrf2 nuclear translocation, increasing GSK3β phosphorylation, decreasing KEAP1 expression, and raising GPX4 levels (44).

Ferroptosis in alveolar epithelial cells is caused by a number of important processes and regulatory variables. One important mechanism that controls redox homeostasis and guards against ferroptosis is the Nrf2/ARE pathway (45). Anti-ferroptotic genes, including SLC7A11 and HO-1, are transcriptionally activated by Nrf2, promoting cellular survival. According to a study on intestinal ischemia-reperfusion (IIR)-induced ALI/ARDS, Nrf2 inhibits oxidative stress and attenuates ferroptosis via controlling the amounts of ferroptosis-related proteins, such as SLC7A11 and GPX4. Additional regulatory elements include PCTR1, circEXOC5, and AUF1. The mRNA-binding protein AUF1 reduces sepsis-associated ALI damage by negatively affecting ATF3 and positively regulating Nrf2, which in turn controls ferroptosis (46). Through the enhancement of ATF3 mRNA degradation and the reduction of GPX4 levels, CircEXOC5 controls the IGF2BP2/ATF3 axis to promote ferroptosis (47). The potential of PCTR1, a protectin compound, as a therapeutic target has been highlighted by its ability to control ferroptosis in alveolar epithelial cells (48).

Ferroptosis inhibitors have been shown in preclinical research to lessen lung damage in ARDS animals. Ferrostatin-1 and Liproxstatin-1, two well-known inhibitors of ferroptosis, for instance, have demonstrated protective benefits in both ARDS and ALI models (49). These inhibitors prevent cell death by lowering lipid peroxidation. In a study that used an LPS-induced ALI model, ferrostatin-1 therapy successfully decreased inflammation and lung damage (50). Likewise, it has been demonstrated that ginseng diol (Px), which is extracted from the root of ginseng, reduces LPS-induced ALI/ARDS via activating the KEAP1/Nrf2/HO-1 pathway (51).

### 3.2 Endothelial cell dysfunction

**Figure 4:**
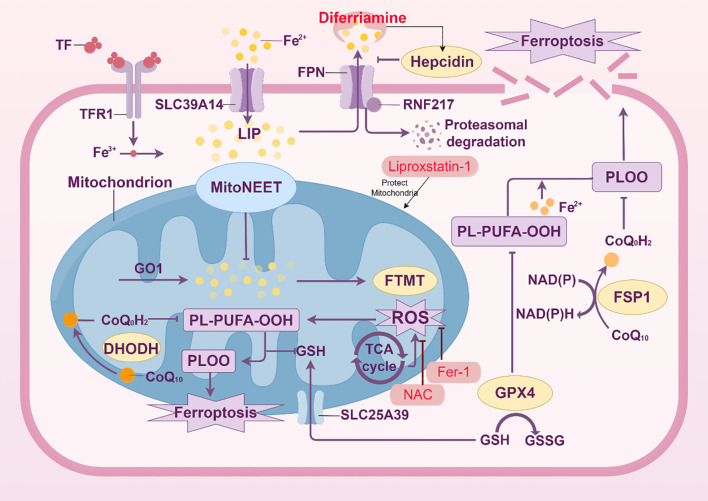
Mechanisms of ferroptosis in endothelial cells. The intricate metabolic and signaling processes involved in ferroptosis, a type of controlled cell death marked by iron-dependent lipid peroxidation, particularly in endothelial cells, are depicted in this picture. Iron buildup triggers ferroptosis, which is controlled by ferroportin (FPN) and hepcidin and mediated by the transferrin receptor (TFR1). Increased labile iron pool (LIP) from iron overload catalyzes the Fenton reaction, which produces lipid reactive oxygen species (ROS) and peroxidation products (such PL-PUFA-OOH). Several important defense mechanisms against ferroptosis are highlighted in the figure. By converting CoQ10 to ubiquinol (CoQoH2), which scavenges ROS, the mitochondrial protein MitoNEET and the ferroptosis suppressor protein 1 (FSP1) have been demonstrated to preserve mitochondria. Lipid peroxide detoxification relies heavily on the glutathione (GSH) system, which includes the enzyme GPX4 and the GSH/GSSG balance. Furthermore, the function of the mitochondrial GSH transporter SLC25A39 in preserving mitochondrial GSH levels is highlighted. The effectiveness of ferroptosis inhibitors, including Liproxstatin-1 and Fer-1, to stop lipid peroxidation and stop cell death is highlighted. On the other hand, it has been demonstrated that ferroptosis inducers such as RSL3 interfere with these defenses. The role of NAD(P)H and the elements of the electron transport chain (such as CoQ10 and DHODH) in preserving redox homeostasis is also depicted in the picture. The balance between pro- and anti-ferroptotic components is highlighted in this picture, which offers a thorough summary of the molecular pathways and regulatory mechanisms that control ferroptosis in endothelial cells.

One characteristic of ARDS is endothelial cell dysfunction, which exacerbates lung injury by disrupting the alveolar-capillary barrier. One important mechanism behind endothelial cell dysfunction in ARDS is ferroptosis, a kind of cell death caused by iron-dependent lipid peroxidation (52) (**Figure 4**).

Iron-dependent lipid peroxidation, which causes lipid peroxides to build up and ultimately cause cell death, is a hallmark of ferroptosis. Because endothelial cells are subjected to severe oxidative stress in ARDS, this mechanism is especially pertinent. ARDS patients have been found to have dysregulated iron metabolism, as evidenced by elevated levels of total and non-heme iron in plasma and bronchoalveolar lavage fluid (BALF) when compared to healthy controls. Ferroptosis’s involvement is reinforced by the recent finding of lipid hydroperoxides in the pulmonary drainage fluid of ARDS patients (53).

Lipid peroxidation, the glutathione (GSH)/GPX4 axis, and iron metabolism comprise the central signaling mechanism of ferroptosis. The Fenton reaction, in which iron catalyzes the production of reactive oxygen species (ROS) that target polyunsaturated fatty acids (PUFAs) in cell membranes, can cause ferroptosis in endothelial cells. Lipid peroxides produced by this mechanism have the potential to cause cell death. One important aspect of lipid peroxidation is the generation of hydroxyl radicals (OH•), which can be fatal to cells if they accumulate excessively (54).

Through an array of procedures, ferroptosis contributes to endothelial cell damage as ARDS progresses (55). Patients with ARDS may have elevated iron levels in their lungs, which may lead to oxidative stress while stimulating lipid peroxidation (56). As a result, the alveolar-capillary barrier collapses, vascular permeability increases, and pulmonary edema develops. Ferroptosis inhibitors, including ferrostatin-1 and lipostatin-1, have been demonstrated in studies that reduce lung damage in ARDS models by decreasing lipid peroxidation and inhibiting cell death. In the meantime, ferroptosis in endothelial cells during ARDS is modulated by a number of regulatory variables and pathways. In order to maintain a redox state of equilibrium and prevent ferroptosis, the transcription factor Nrf2 is essential. Anti-ferroptotic genes, including SLC7A11 and HO-1, which support cellular survival, have their transcription activated by Nrf2. According to a study on LPS-induced ARDS, Nrf2 inhibits oxidative stress and attenuates ferroptosis via controlling the amounts of ferroptosis-related proteins, such as SLC7A11 and GPX4. Additional regulatory elements include PCTR1 (48), circEXOC5 (47), and AUF1 (46). The mRNA-binding protein AUF1 reduces sepsis-associated ALI damage by negatively affecting ATF3 and positively regulating Nrf2, which in turn controls ferroptosis. Through the enhancement of ATF3 mRNA degradation and the reduction of GPX4 levels, CircEXOC5 controls the IGF2BP2/ATF3 axis to promote ferroptosis (47).

### 3.3 Fibroblast

The survival controls for ARDS fibroblasts differ from those for healthy lung fibroblasts. They have a higher fibroproliferative capacity and can divide *in vitro* without the need for extra growth factors. If this increased proliferative ability is not appropriately controlled, it may result in excessive fibrosis. During the resolution phase of ARDS, ferroptosis has been identified as a method to limit fibroblast proliferation and eliminate surplus cells (57).

Ferroptosis in fibroblasts is caused by a number of important processes and regulatory variables. The control of lipid peroxidation and iron metabolism is one important mechanism. Cell death may result from lipid peroxidation brought on by iron excess. Enzymes like glutathione peroxidase 4 (GPX4), which lowers lipid hydroperoxides and inhibits lipid peroxidation, control this process. Ferroptosis results from increased lipid peroxidation caused by GPX4 deficiency. The tumor suppressor p53 and its downstream targets regulate ferroptosis, which is another significant mechanism. By upregulating genes related to lipid peroxidation and iron absorption, p53 can cause ferroptosis. Furthermore, fibroblasts are shielded against ferroptosis by the Nrf2/ARE pathway, which controls redox homeostasis. Anti-ferroptotic genes, including SLC7A11 and HO-1, are transcriptionally activated by Nrf2, promoting cellular survival. More information on the function of ferroptosis in fibroblasts during ARDS has been revealed by recent investigations. One study showed, for example, that fibroblasts from ARDS patients are more prone to ferroptosis than normal fibroblasts themselves (9). Higher iron and lipid peroxidation markers are linked to this heightened vulnerability. Inhibiting ferroptosis in fibroblasts has been demonstrated in another study to decrease fibroproliferation and enhance lung function in ARDS animals (58).

### 3.4 Immune cell dysregulation

**Figure 5:**
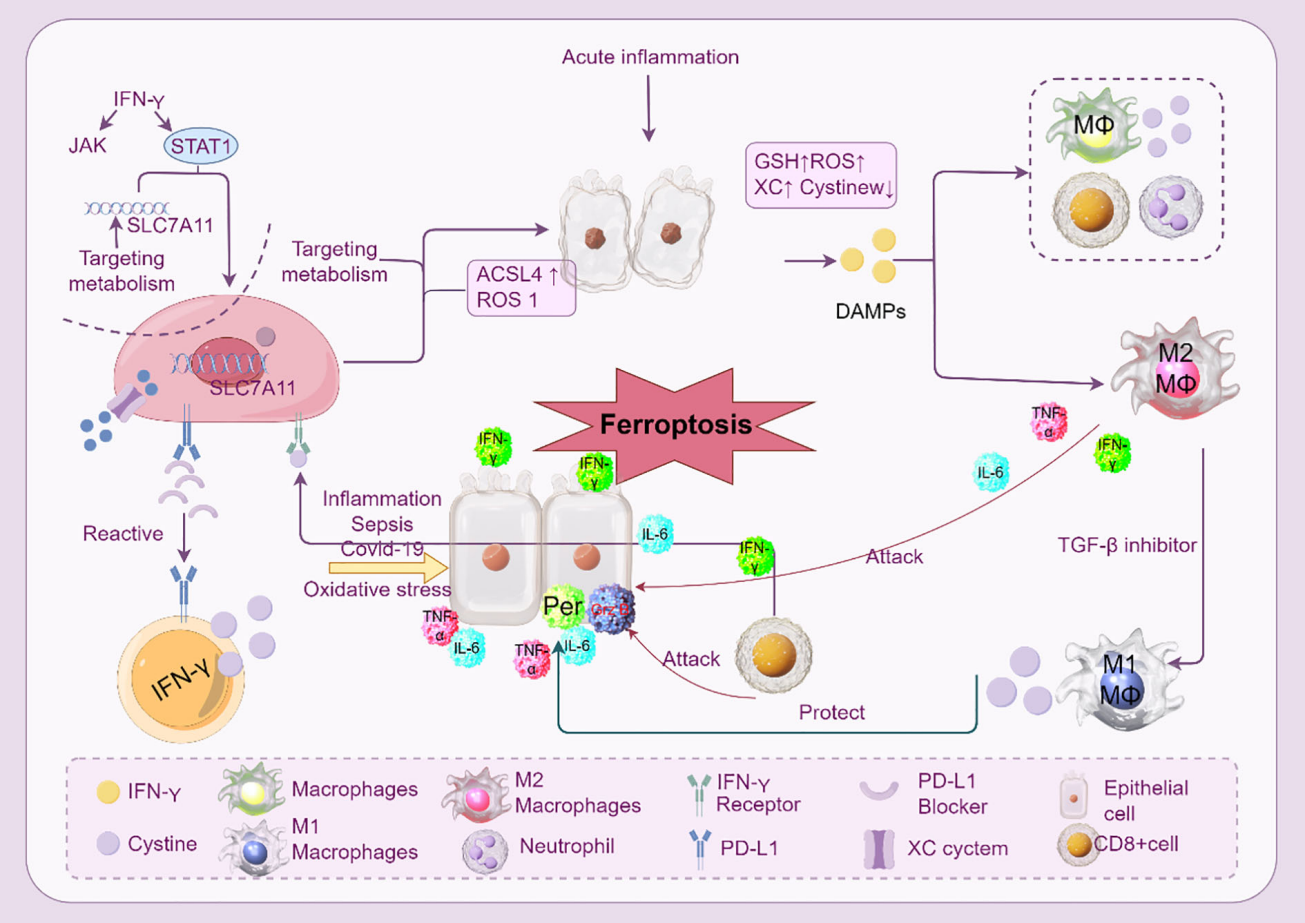
Ferroptosis and immune cells in ARDS. The interaction between immune cells and ferroptosis during acute inflammation and sepsis-induced ARDS is depicted. Numerous immune cells and cytokines control ferroptosis, which is typified by iron-dependent lipid peroxidation. By producing cytokines like IL-6 and TNF-α, macrophages (M1 and M2) are essential in controlling ferroptosis. By downregulating the cystine-glutamate antiporter (SLC7A11) and upregulating the generation of ROS and ACSL4, IFN-γ, which is generated by CD8+ T cells, can cause ferroptosis in macrophages. By producing ROS and DAMPs, neutrophils also aid in ferroptosis. The image emphasizes the role of metabolic pathways (like GSH and ROS) and the consequences of using inhibitors to target these pathways (such ferroptosis inhibitors and SLC7A11 targeting). Furthermore, the function of epithelial cells in triggering ferroptosis via receptor blockage and PD-L1 expression is illustrated.

Immune cells, including macrophages, neutrophils, T cells, and B cells, play a dual role in ARDS pathogenesis. Ferroptosis in these cells disrupts immune homeostasis and contributes to inflammation and tissue injury (**Figure 5**).

#### 3.4.1 Macrophages

In response to environmental stimuli, macrophages can polarize into different phenotypes, demonstrating their extraordinary adaptability. M1 (classically activated) and M2 (alternatively activated) macrophages are the two primary phenotypes. While M2 macrophages are anti-inflammatory and support tissue healing and inflammation, M1 macrophages are pro-inflammatory and participate in eliminating infections and tissue damage. By changing the cellular environment and the accessibility of iron, which is essential for macrophage activity, ferroptosis might affect macrophage polarization. In diseases like ARDS, iron excess can exacerbate tissue damage by skewing macrophage polarization toward a pro-inflammatory M1 phenotype. On the other hand, suppressing ferroptosis might enhance an M2 phenotype that is anti-inflammatory, which may help decrease tissue damage and inflammation (59).

Depletion of glutathione peroxidase 4 (GPX4), an enzyme that lowers lipid hydroperoxides and inhibits lipid peroxidation, is the main cause of ferroptosis in macrophages (60). Cell death results from increased lipid peroxidation caused by GPX4 deficiency. The excessive accumulation of iron, which triggers the Fenton reaction and produces reactive oxygen species (ROS), makes this process more severe. Macrophages in ARDS are more vulnerable to ferroptosis because they are subjected to greater amounts of oxidative stress and iron overload. Ferroptotic macrophages can intensify the inflammatory response by releasing pro-inflammatory cytokines and damage-associated molecular patterns (DAMPs). For example, ferroptotic macrophages have the ability to generate HMGB1, a DAMP that triggers the release of pro-inflammatory cytokines, including IL-1β and IL-18, and activates the inflammasome (61).

By increasing inflammation and blocking tissue healing, ferroptosis in macrophages in ARDS increases the severity of lung damage. Ferroptotic macrophages can trigger a systemic inflammatory response by releasing DAMPs and pro-inflammatory cytokines that can activate the inflammasome. This contributes to the pathophysiology of ARDS by generating a self-amplifying loop of inflammation. Furthermore, ferroptosis may disrupt macrophage function by decreasing their capacity to eliminate infections and their phagocytic activity. This may result in a persistent inflammatory reaction and a delayed recovery from lung damage. Research has demonstrated that in ARDS models, preventing macrophage ferroptosis may minimize tissue damage and inflammation (62).

A potential treatment approach to reduce inflammation and tissue damage in ARDS is to target ferroptosis in macrophages. It has been demonstrated that ferroptosis inhibitors, such as ferrostatin-1 (Fer-1) and lipostatin-1, lower lipid peroxidation and arrest macrophage death. These inhibitors may improve ARDS outcomes by regulating the inflammatory response (63). Furthermore, enhancing cellular antioxidant defenses by targeting the Nrf2 pathway may offer a viable strategy for reducing macrophage dysfunction and improving ARDS outcomes (64). Anti-ferroptotic genes, including SLC7A11 and HO-1, which support cellular survival, have their transcription activated by Nrf2.

#### 3.4.2 Neutrophils

The most prevalent kind of white blood cells, neutrophils, are among the first to arrive at areas of inflammation and infection (65). They play a crucial role in the phagocytosis of pathogens, the release of reactive oxygen species (ROS), and antimicrobial peptides. However, by releasing oxidants, proteases, and other inflammatory mediators, neutrophils can cause tissue damage in diseases like ARDS. Numerous triggers, such as infection, ischemia-reperfusion injury, and exposure to pro-inflammatory cytokines, can cause neutrophils to undergo ferroptosis. Increased iron absorption, lipid peroxidation, and the buildup of lipid peroxides, which results in cell death, are the hallmarks of the process. This type of cell death differs from necrosis and apoptosis in that it involves particular morphological changes and metabolic processes (66).

Numerous important pathways and regulatory variables are involved in the molecular mechanisms that underlie neutrophil ferroptosis. The reduction of glutathione peroxidase 4 (GPX4), an enzyme that lowers lipid hydroperoxides and inhibits lipid peroxidation, is one important mechanism (67). Cell death results from increased lipid peroxidation caused by GPX4 deficiency. Furthermore, iron buildup exacerbates oxidative stress and lipid peroxidation by catalyzing the Fenton reaction, which produces ROS. The Nrf2/ARE pathway, which controls redox homeostasis and fights against ferroptosis, is another regulatory component. Anti-ferroptotic genes, including SLC7A11 and HO-1, are transcriptionally activated by Nrf2, promoting cellular survival. These pathways are crucial for neutrophil survival and function since blocking them can make individuals more susceptible to ferroptosis (66).

Neutrophils are frequently detected in a highly active state in ARDS, which exacerbates lung injury and causes the release of pro-inflammatory cytokines. Neutrophils that undergo ferroptosis may die and emit damage-associated molecular patterns (DAMPs), which intensifies the inflammatory response (68). For example, HMGB1, a DAMP that triggers the inflammasome and promotes the release of pro-inflammatory cytokines including IL-1β and IL-18, can be released by ferroptotic neutrophils. This exacerbates ARDS by generating a self-reinforcing cycle of inflammation. Furthermore, ferroptosis can hinder neutrophil function by decreasing their capacity to eliminate infections and engage in phagocytic activity. This may result in a protracted inflammatory reaction and a delayed recovery from lung damage. In ARDS models, studies have demonstrated that preventing neutrophil ferroptosis can lessen tissue damage and inflammation (69).

#### 3.4.3 T cells

The inflammatory response and tissue damage seen in ARDS are largely caused by T cells, which are essential elements of the adaptive immune system (70). Here, we provide an overview of the connection between ferroptosis and T cell function, with particular attention to the roles that several T cell subtypes, including Th1, Th2, Th17, Th22, Treg, γδ T cells, effector T cells, and memory T cells, in ARDS (70).

Pro-inflammatory Th1 cells generate cytokines including TNF-α and IFN-γ, which are essential for combating intracellular infections. Through the secretion of these cytokines, Th1 cells in ARDS may be involved in tissue destruction. Th1 cells that undergo ferroptosis may perish and emit damage-associated molecular patterns (DAMPs), which intensifies the inflammatory response. Research has demonstrated that in ARDS models, preventing Th1 cell ferroptosis can lessen tissue damage and inflammation (71). Th2 cells are known to produce cytokines, including IL-4, IL-5, and IL-13, and are important in the immune response towards external infections. Th2 cells are useful in tissue regeneration and inflammation resolution in ARDS. Th2 cells that undergo ferroptosis might function less effectively and cause a prolonged inflammatory response (72). It may be possible to sustain Th2 cells’ anti-inflammatory properties and enhance ARDS results by preventing ferroptosis (71). Th17 cells are essential for the immune response against extracellular bacteria and fungi because they generate IL-17. Th17 cells can cause tissue damage in ARDS by releasing IL-17, which encourages neutrophil activation and recruitment. Th17 cell ferroptosis can result in DAMP release and cell death, which exacerbates inflammation (73, 74). Th17 cell ferroptosis inhibition could mitigate tissue damage along with improving ARDS outcomes (72). IL-22, which is important in barrier function and tissue repair, is produced by Th22 cells, a subgroup of T helper cells. Th22 cells may assist in tissue regeneration and inflammation resolution in ARDS. Th22 cells that undergo ferroptosis have reduced function and cause a lengthy inflammatory response (75). A distinct subgroup of T cells known as γδ T cells is capable of identifying and reacting to a broad variety of infections. γδ T cells may help in tissue healing and the immune response in ARDS. A protracted inflammatory response can result from ferroptosis in γδ T cells, which can affect their function (76).

Preventing excessive inflammation and preserving immunological tolerance depend on Treg cells. Treg cells may assist in tissue healing and inflammatory response modulation in ARDS (77). Treg cell ferroptosis can affect the extent to which they function and cause unchecked inflammation. Treg cell ferroptosis inhibition may preserve immunological tolerance and enhance ARDS outcomes (70).

Activated T cells that have undergone differentiation to carry out specific activities, such as cytotoxicity or cytokine production, are known as effector T cells (78). Effector T cells can cause tissue damage in ARDS by releasing cytotoxic and pro-inflammatory cytokines (79). Effector T cell ferroptosis can result in DAMP release and cell death, which intensifies the inflammatory response. Reducing tissue damage and improving outcomes in ARDS may be achieved by inhibiting effector T cell ferroptosis.

#### 3.4.4 B cells

The adaptive immune system’s main constituents, B cells, are essential for the tissue damage and immunological response seen in ARDS (80). Depletion of glutathione peroxidase 4 (GPX4), an enzyme that lowers lipid hydroperoxides and inhibits lipid peroxidation, is the main cause of ferroptosis in B cells (81). Cell death results from increased lipid peroxidation caused by GPX4 deficiency. Furthermore, iron buildup exacerbates oxidative stress and lipid peroxidation by catalyzing the Fenton reaction, which produces reactive oxygen species (ROS).

Generating an adaptive immune response requires naive B cells, which are the progenitors of effector and memory B cells. Naive B cells may become fewer in number and less able to react to antigens if ferroptosis is induced in them. Since effector B cells are necessary for the creation of antibodies and other immunological processes, this can subsequently impair the immune response as a whole (82). According to studies, p53-mediated reactions and the stimulation of polyamine metabolism can cause ferroptosis in naive B cells. Spermidine/spermine N (1)-acetyltransferase (SAT1) is upregulated in this process, and it interacts with p53-mediated ferroptotic reactions to promote polyamine metabolism (83). Effector B cells are activated B cells that have undergone differentiation to carry out particular tasks, such as producing antibodies. By releasing pro-inflammatory cytokines and antibodies, effector B cells in ARDS can cause tissue damage. DAMPs are released when effector B cells undergo ferroptosis, which can result in cell death and intensify the inflammatory response (84). Inhibiting effector B cell ferroptosis may reduce tissue damage and enhance ARDS results. When re-exposed to a pathogen, memory B cells, the long-lived B cells, offer a strong and quick defense. Memory B cells can support tissue healing and the immune response in ARDS (81). A subpopulation of B cells known as Bregs is essential for preserving immunological tolerance and limiting excessive inflammation. Bregs with ferroptosis may have impaired function and uncontrolled inflammation. According to research, Breg activity can be inhibited by downregulating the expression of the thioredoxin (TXN) gene, which encourages the development of pro-inflammatory B cells and causes systemic inflammation (85). On the other hand, in illness models, elevated TXN expression can reduce lung tissue damage and raise survival rates (82). Ferroptosis in B cells is controlled by similar signaling mechanisms (81).

### 3.5 Other cells

For lung regeneration and repair, pulmonary endogenous stem cells are essential. These cells are essential for preserving lung homeostasis because they have the ability to develop into a variety of lung cell types, such as endothelial and alveolar epithelial cells (86). Significant damage is done to the lung tissue in ARDS, and tissue regeneration and repair depend on endogenous stem cell activation. According to recent research, ARDS patients frequently exhibit downregulated expression levels of pulmonary endogenous stem cells, which may hinder the lung’s capacity for self-healing. Multipotent stromal cells called mesenchymal stem cells (MSCs) have the ability to differentiate into a variety of cell types, such as adipocytes, osteoblasts, and chondrocytes. Because of their immunomodulatory and tissue-repair capabilities, MSCs have demonstrated potential in the treatment of ARDS. MSCs can release growth factors and anti-inflammatory cytokines, which aid in tissue repair and inflammation reduction in the lungs (87). Furthermore, MSCs can improve the viability and function of injured lung cells by transferring mitochondria to them. One important way that MSCs lessen lung damage in ARDS is by this mitochondrial translocation (88).

MSCs, ferroptosis, and pulmonary endogenous stem cells interact in a complicated and multidimensional way. Both MSCs and pulmonary endogenous stem cells have the ability to alter the lung’s inflammatory milieu, which can affect how vulnerable lung cells are to ferroptosis. MSCs, for instance, have the ability to release substances that prevent ferroptosis, shielding lung cells from iron-dependent lipid peroxidation (89). Furthermore, pulmonary endogenous stem cell activation can encourage tissue healing and lessen the need for ferroptosis-induced cell replacement. Novel therapeutic approaches for ARDS may be offered by focusing on the inhibition of ferroptosis of MSCs and pulmonary endogenous stem cells. Enhancing the activity of MSCs and pulmonary endogenous stem cells, for example, may encourage lung healing and lessen the severity of ARDS. Furthermore, preventing ferroptosis may prevent the death of vital lung cells, maintaining lung function. MSC therapy for ARDS has demonstrated encouraging outcomes in recent clinical trials, underscoring the cells’ capacity to reduce inflammation and encourage tissue repair (68).

## 4 Ferroptosis and ARDS progression

### 4.1 Oxidative stress and ROS amplification

Oxidative stress is a hallmark of ARDS and plays a pivotal role in driving ferroptosis. The excessive production of ROS during ARDS creates an environment conducive to ferroptotic cell death. In alveolar epithelial cells and endothelial cells, mitochondrial dysfunction serves as a major source of ROS. Damage to the mitochondrial electron transport chain increases ROS leakage, further amplifying oxidative stress (12). This ROS overproduction, compounded by elevated iron levels, enhances lipid peroxidation, a key feature of ferroptosis.

Lipid peroxidation forms a feedback loop that exacerbates ferroptosis and ARDS progression. Lipid hydroperoxides accumulate in the cellular membranes of lung cells, destabilizing the barrier functions of alveolar epithelial and endothelial cells. The inability of antioxidant systems, such as GPX4-dependent pathways, to counteract ROS amplifies ferroptotic damage (90). This cycle of ROS generation and lipid peroxidation perpetuates oxidative injury, worsening inflammation, vascular permeability, and respiratory dysfunction in ARDS patients (91).

### 4.2 Iron overload and dysregulated metabolism

Iron overload is a critical factor that drives ferroptosis and worsens ARDS pathology. In ARDS, disrupted iron homeostasis leads to the accumulation of free iron, increasing the availability of Fe2^+^ for the Fenton reaction. This reaction generates hydroxyl radicals, a highly reactive form of ROS, which accelerates lipid peroxidation and cell death. Elevated levels of ferritin and transferrin, markers of iron dysregulation, have been observed in clinical ARDS cases, linking iron metabolism to disease severity (12).

Mitochondrial dysfunction exacerbates iron-driven ferroptosis by impairing the organelle’s ability to sequester and regulate iron levels. Mitochondrial iron accumulation further disrupts redox homeostasis, amplifying oxidative stress and contributing to persistent cellular injury (92). Dysregulated iron metabolism not only promotes ferroptosis but also disrupts essential metabolic processes required for tissue repair, highlighting its role in ARDS progression (53).

### 4.3 Fibrosis and long-term outcomes

The chronic inflammation and unresolved ferroptosis associated with ARDS contribute significantly to lung fibrosis and long-term complications. Persistent ferroptosis in alveolar and endothelial cells triggers pro-fibrotic signaling pathways, such as those involving TGF-β and connective tissue growth factor (CTGF), which drive ECM deposition and fibroproliferation. This fibroproliferative response compromises lung elasticity, impairs alveolar regeneration, and leads to irreversible remodeling of lung tissue (93).

Unresolved ferroptosis also hinders the recovery of injured lung cells. By depleting populations of functional alveolar epithelial cells, ferroptosis prevents the repair of the alveolar-capillary barrier, leaving the lung vulnerable to further injury (94). In survivors of ARDS, this impaired regenerative capacity manifests as pulmonary fibrosis, characterized by reduced lung compliance and chronic respiratory dysfunction. These long-term consequences underscore the need for therapeutic strategies that can effectively mitigate ferroptosis and its downstream effects.

## 5 Therapeutic targeting of ferroptosis in ARDS

**Figure 6:**
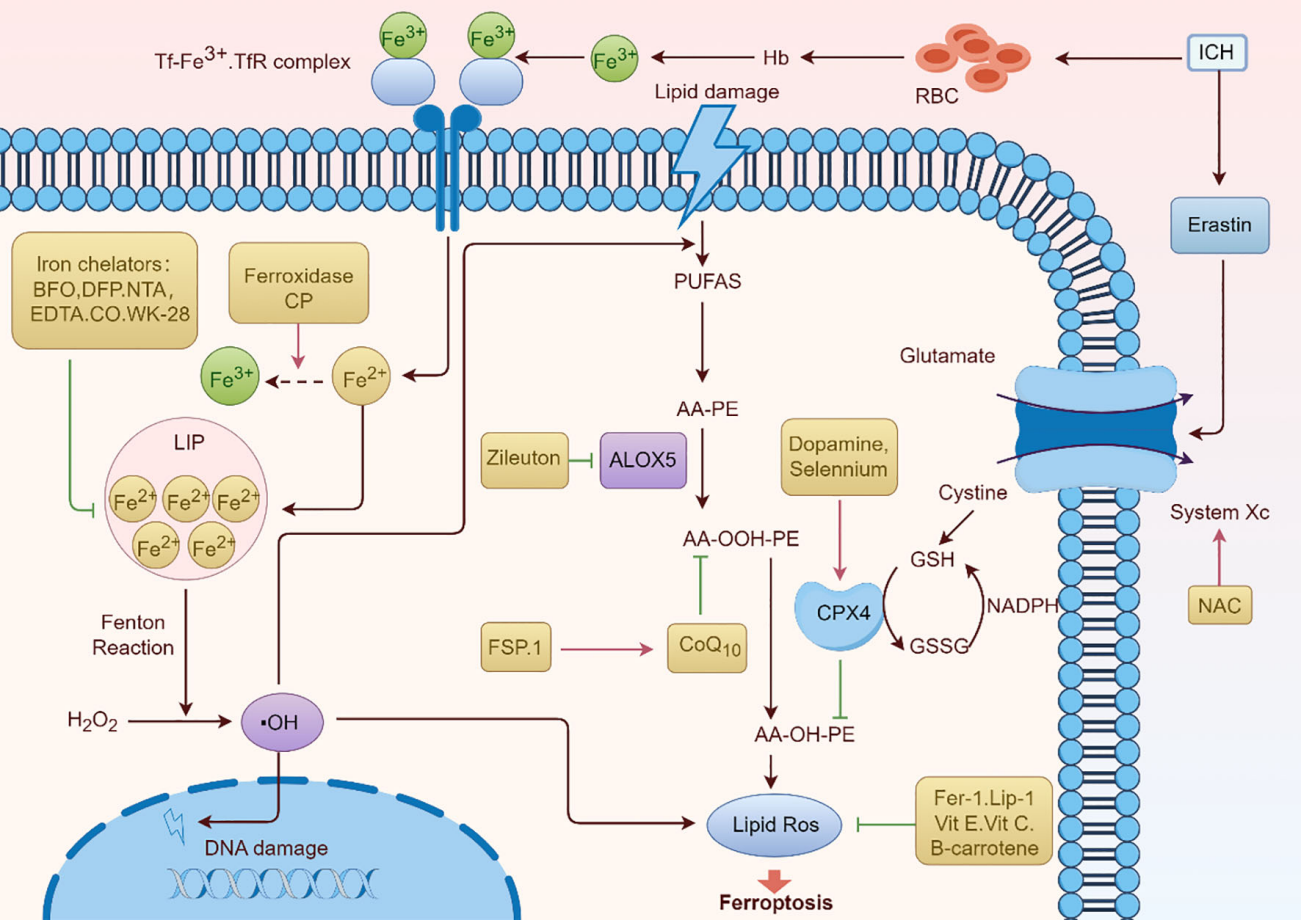
Ferroptosis pathway and potential therapeutic drugs for ARDS. The processes and mechanisms underlying ferroptosis, a type of controlled cell death marked by iron-dependent lipid peroxidation, are shown in **Figure 6**. Important elements like the Tf-Fe3+.TfR complex, which promotes iron absorption, and the function of hemoglobin (Hb) and red blood cells (RBC) in iron-related processes are highlighted in the image. It also demonstrates how lipid damage is involved and how iron chelators (like EDTA and DFP) and antioxidants (such NAC, vitamin E, and vitamin C) can protect against it. The figure depicts the contribution of enzymes such as ALOX5 and FSP.1 to lipid peroxidation, as well as the roles of glutathione (GSH) and NADPH in antioxidant defense. A thorough summary of the molecular processes underpinning ferroptosis is given in this graphic.

Given the central role of ferroptosis in ARDS pathogenesis, therapeutic strategies targeting ferroptotic pathways offer promising avenues to mitigate disease severity and improve outcomes. Interventions aimed at regulating oxidative stress, restoring antioxidant defenses, chelating iron, and preventing lipid peroxidation are at the forefront of these efforts (**Figure 6**).

### 5.1 Antioxidants and ROS inhibitors

Oxidative stress drives ferroptosis and contributes to the lung injury observed in ARDS. Antioxidants and ROS inhibitors aim to reduce oxidative damage by restoring redox balance. NAC serves as a precursor for glutathione (GSH) synthesis, a critical antioxidant that neutralizes ROS and protects cells from lipid peroxidation. By replenishing intracellular GSH levels, NAC helps restore the antioxidant capacity of lung cells and mitigate oxidative stress-induced ferroptosis (95, 96). MitoTEMPO: This mitochondrial-targeted antioxidant scavenges mitochondrial ROS, which play a significant role in initiating ferroptosis. MitoTEMPO’s targeted action protects mitochondrial function and reduces lipid ROS formation, safeguarding both alveolar epithelial and endothelial cells from ferroptotic cell death (97). Emerging antioxidants designed to specifically target ROS in the lung microenvironment may provide further benefits in managing ARDS.

### 5.2 GPX4 activators

As a key regulator of ferroptosis, glutathione peroxidase 4 (GPX4) plays a critical role in preventing lipid peroxidation and maintaining cellular homeostasis. GPX4 activators have shown promise in halting ferroptosis in preclinical studies. Ferrostatins (e.g., Fer-1)is the small molecules that inhibit lipid peroxidation by stabilizing GPX4 activity, thus preventing the ferroptotic death of lung cells (98). Fer-1 has been demonstrated to protect against oxidative injury in lung tissues by limiting the accumulation of toxic lipid hydroperoxides (99). RSL3 is a GPX4 inhibitor that drives ferroptosis by reducing the enzyme’s activity. Therapeutics that block RSL3-mediated GPX4 inactivation may provide a targeted approach to preserving antioxidant capacity in ARDS patients, reducing the extent of ferroptosis-driven lung damage (13).

### 5.3 Iron chelators

Iron overload exacerbates ferroptosis by fueling ROS production and lipid peroxidation, making iron chelation a viable therapeutic approach. Deferoxamine is the first one. It is clinically established iron chelator binds excess free iron, thereby reducing its availability for the Fenton reaction and subsequent ROS generation. Deferoxamine has demonstrated efficacy in preclinical models of ARDS, where it mitigates iron-driven oxidative damage and inflammation (100). Another potent iron chelator, deferiprone, offers the advantage of oral administration. By limiting iron availability, it curtails ferroptotic damage in lung cells and supports redox balance during ARDS progression (101). Iron chelation therapies may also modulate downstream pro-inflammatory and pro-fibrotic effects linked to ferroptosis, underscoring their therapeutic potential in ARDS.

### 5.4 Targeting lipid peroxidation

Preventing lipid peroxidation is critical to halting ferroptosis in ARDS, as peroxidized lipids drive cellular dysfunction and death. Acyl-CoA synthetase long-chain family member 4 (ACSL4) facilitates the incorporation of PUFAs into membrane phospholipids, increasing their susceptibility to peroxidation. Inhibitors of ACSL4 reduce this vulnerability, preserving membrane integrity and protecting lung cells from ferroptotic injury (102). Therefore, ACSL4 inhibitor might be a potential therapeutic drug for ARDS. There is another one targeting lipid ROS scavengers. Compounds like liproxstatin-1 neutralize lipid peroxides, preventing their accumulation and halting ferroptosis. Liproxstatin-1 has shown potential in preclinical models of lung injury by reducing oxidative damage, inflammation, and vascular permeability (103). Future research may focus on optimizing the delivery of lipid ROS scavengers to the lungs, ensuring precise targeting of injured tissues while minimizing systemic side effects (**Table 1**).

**Table 1:** Ferroptosis inhibitors and therapeutic potential in ARDS.

Inhibitor name	Dose	Cell/Animal model	Targeted cells	Molecular mechanisms
Dipyridamole (DIPY)	Not specified	LPS-induced acute lung injury mouse model, CLP-induced sepsis mouse model, Human airway organoids (HAOs)	Lung epithelial and endothelial cells	Downregulates heme oxygenase 1 (HMOX1) by binding to and activating superoxide dismutase 1 (SOD1), inhibiting the CREB1/HMOX1 pathway
Ferrostatin-1 (Fer-1)	2.5 μM/kg	LPS-induced ARDS mouse model	Alveolar epithelial cells	Inhibits ferroptosis, reduces lipid peroxidation, and protects against lung injury
Ferrostatin-1 (Fer-1)	Not specified	Sepsis mouse model, MLE-12 cells	Alveolar epithelial cells	Increases GPX4 expression, reduces lipid peroxidation, and inhibits ferroptosis
Hepcidin	Not specified	LPS-induced ARDS mouse model	Lung cells	Upregulates ferritin heavy chain (FTH) to reduce labile iron pool (LIP) and inhibit ferroptosis
Liproxstatin-1	Not specified	LPS/IL-13-induced bronchial epithelial cell injury and neutrophilic asthma mouse model	Bronchial epithelial cells	Inhibits ferroptosis and reduces inflammation
Panaxydol	Not specified	LPS-induced acute lung injury mouse model	Lung cells	Activates Keap1-Nrf2/HO-1 pathway to inhibit ferroptosis

## 6 Future directions and challenges

Despite significant advancements in understanding the role of ferroptosis in ARDS, several challenges remain in translating these findings into effective clinical strategies. Future research must address the gaps in biomarkers, subphenotype-specific mechanisms, and the integration of ferroptosis-targeted therapies into standard care.

### 6.1 Biomarkers of ferroptosis in ARDS

The identification of reliable biomarkers is critical for diagnosing ferroptosis-driven ARDS, stratifying patients for treatment, and monitoring therapeutic responses. Potential candidates include lipid peroxidation byproducts, such as malondialdehyde (MDA) and 4-hydroxynonenal (4-HNE), which are indicative of oxidative damage (104). Additionally, the measurement of glutathione peroxidase 4 (GPX4) activity and reduced glutathione (GSH) levels may serve as functional markers of ferroptosis susceptibility (105). Advanced lipidomic profiling and transcriptomic approaches could further refine biomarker discovery, enabling the differentiation of ferroptosis from other cell death pathways, such as apoptosis and necroptosis, in ARDS. The establishment of standardized protocols for biomarker assessment in clinical settings remains an ongoing challenge.

### 6.2 Role of ferroptosis in ARDS subphenotypes

ARDS is a heterogenous syndrome with distinct subphenotypes, including hyperinflammatory and hypoinflammatory forms, which differ in pathophysiology, clinical presentation, and response to treatment. Investigating whether ferroptosis contributes differentially to these subphenotypes could provide valuable insights for personalized therapies. For instance, hyperinflammatory ARDS, characterized by excessive cytokine release and oxidative stress, may exhibit higher ferroptosis activity than hypoinflammatory ARDS. Identifying these differences through advanced molecular and imaging techniques could help tailor ferroptosis-targeted interventions to specific subphenotypes, optimizing therapeutic efficacy. Further exploration of animal models and patient-derived organoid systems may facilitate these investigations.

### 6.3 Combining ferroptosis inhibition with standard therapies

Integrating ferroptosis-targeted therapies with existing ARDS interventions, such as corticosteroids, immunomodulators, or mechanical ventilation, presents both opportunities and challenges. While corticosteroids may alleviate inflammation, they could interact with ferroptosis inhibitors in unpredictable ways, necessitating careful evaluation of combinatorial effects. Additionally, mechanical ventilation, a cornerstone of ARDS management, may influence ferroptosis through ventilation-induced oxidative stress, further complicating treatment strategies. Future clinical trials should investigate the safety, timing, and dosing of ferroptosis inhibitors when combined with standard-of-care therapies. Preclinical studies exploring the synergistic effects of such combinations are also essential for advancing this approach.

Efforts to optimize drug delivery systems, such as nanoparticle-based carriers, may enhance the precision and efficacy of ferroptosis inhibitors while minimizing off-target effects. Furthermore, addressing the challenges of patient heterogeneity, drug resistance, and adverse effects will be critical for the successful translation of ferroptosis-targeted therapies into clinical practice.

## 7 Conclusion

Maintaining a balance between cell survival and regulated cell death pathways is fundamental to preserving tissue integrity and immune homeostasis in ARDS. While apoptosis plays a protective role in clearing infected cells in a controlled manner, its dysregulation can lead to immune overactivation, exacerbating inflammation and tissue damage. In contrast, ferroptosis has emerged as a key driver of ARDS pathogenesis, contributing to epithelial and endothelial cell injury, oxidative stress, and immune dysfunction.

Therapeutic targeting of ferroptosis holds great promise for mitigating ARDS severity. Antioxidants and ROS inhibitors reduce oxidative damage, iron chelators limit iron-mediated toxicity, and GPX4 activators preserve cellular antioxidant capacity, collectively offering a multifaceted approach to suppressing ferroptosis in the injured lung. However, several challenges remain, including the identification of reliable biomarkers, understanding subphenotype-specific mechanisms, and integrating ferroptosis inhibitors into existing therapeutic frameworks.

Future research should prioritize addressing these challenges to enhance the clinical translation of ferroptosis-targeted therapies. Combining these innovative approaches with personalized medicine strategies may not only improve outcomes for ARDS patients but also provide valuable insights into the broader implications of ferroptosis in critical illness. By bridging gaps in knowledge and practice, therapeutic targeting of ferroptosis offers the potential to transform ARDS management and improve patient survival.
